# Adipose-Derived Exosomes: mediators of crosstalk between Adipose tissue and cancer

**DOI:** 10.1080/15384047.2025.2547564

**Published:** 2025-08-16

**Authors:** Changjian Wang, Zhikun Zheng, Chuangyan Wu, Dan Zhang, Yangchenxi Wang, Sheng Zhang, Geng Wang, Rui Zhou

**Affiliations:** a Department of Thoracic Surgery, Union Hospital, Tongji Medical College, Huazhong University of Science and Technology, Wuhan, China; b Cancer Center, Union Hospital, Tongji Medical College, Huazhong University of Science and Technology, Wuhan, China; c Institute of Radiation Oncology, Union Hospital, Tongji Medical College, Huazhong University of Science and Technology, Wuhan, China; d Hubei Key Laboratory of Precision Radiation Oncology, Wuhan, China; e Department of Gastrointestinal Surgery, Union Hospital, Tongji Medical College, Huazhong University of Science and Technology, Wuhan, China

**Keywords:** Adipose-derived exosomes, cancer, adipose tissue, tumor microenvironment, miRNA, exosome-based therapy

## Abstract

Adipose-derived exosomes (ADEs), a subtype of extracellular vesicles, are critical mediators of communication between adipose tissue and tumors, playing pivotal roles in cancer progression and therapeutic response. These nanoscale vesicles carry microRNAs, proteins, and lipids that influence tumor cell proliferation, migration, metastasis, and immune modulation. The dual functions of ADEs – both in promoting and suppressing tumorigenesis – are largely dependent on their cellular origin, molecular cargo, and the characteristics of the tumor microenvironment. Recent studies have identified ADEs as potential diagnostic biomarkers, therapeutic targets, and drug delivery platforms, offering promising avenues for precision oncology. However, significant challenges – such as biological heterogeneity, lack of standardization in production, concerns regarding efficacy and safety, and regulatory constraints – continue to hinder their clinical translation. This review aimed to explore the multifaceted roles of ADEs in cancer pathogenesis, their therapeutic potential, and current limitations, providing insights to guide future research and clinical applications.

## 1. Introduction

Adipose tissue (AT), previously considered a passive energy reservoir, has been redefined as a dynamic endocrine organ actively involved in the regulation of a wide range of physiological and pathological processes.^1^ This multifunctional tissue comprises a complex multicellular ecosystem composed of mature adipocytes, adipose-derived stem cells (ADSCs), pre-adipocytes, immune cells (such as macrophages, lymphocytes, and myeloid cells), vascular-associated cells (such as pericytes, endothelial cells, and smooth muscle cells), and structural fibroblasts. These diverse cellular populations coordinate metabolic homeostasis and immune modulation through highly regulated and intricate intercellular communication networks.^2^

AT exhibits a remarkable secretory capacity, releasing a broad spectrum of bioactive mediators – including adipokines, cytokines, and extracellular vesicles (EVs) – as part of its secretome.^3^ Among these, exosomes, a well-defined subtype of EVs (30–150 nm in diameter with a phospholipid bilayer), have emerged as central components in the study of intercellular communication. These nanoscale vesicles are ubiquitously present in various biological fluids, including blood plasma, saliva, breast milk, cerebrospinal fluid, urine, and semen.^4^ Their intrinsic biostability, conferred by topological protection, along with their capacity for systemic signaling, underscores their critical role as biological messengers.^5^ Emerging evidence has demonstrated the involvement of adipose-derived exosomes (ADEs) in various pathophysiological processes, including the promotion of angiogenesis,^6,7^ modulation of immune responses,^8,9^ regulation of cellular migration,^10^ and control of bone remodeling through osteogenic differentiation signaling pathways.^11^ These functions are mediated by the selective loading of biologic cargo – such as microRNAs (miRNAs), adipokines, and metabolic enzymes – thereby establishing AT as a systemic signaling hub.^12^

All cell types within AT secrete exosomes, forming a complex network of ADEs. However, the characteristics and biological functions of these exosomes are significantly influenced by their specific cellular origins and depot locations.^13^ ADEs are primarily secreted by mature adipocytes and ADSCs, each exhibiting distinct regulatory roles in cancer biology. ADEs are typically enriched with pro-inflammatory cytokines such as interleukin (IL)-1β, IL-6, IL-8, chemokine (C-C motif) ligand 2 (CCL2), CCL5, and tumor necrosis factor-alpha (TNF-α), which promote cancer cell proliferation and migration by activating the mitogen-activated protein kinase/extracellular signal-regulated kinase, Janus kinase 2/signal transducer and activator of transcription 3 (STAT3), and phosphatidylinositol 3-kinase/protein kinase B (PI3K/AKT) pathways.^14,15^ By contrast, ADSC-derived exosomes more frequently exert tumor-suppressive functions. For example, exosomal miR-34c inhibits β-catenin signaling and epithelial – mesenchymal transition, thereby enhancing apoptosis and increasing radiosensitivity in nasopharyngeal carcinoma cells.^16,17^ Nonetheless, certain ADSC-derived exosomes, such as those containing miR-21, may contribute to tumor progression by downregulating PTEN expression and promoting macrophage polarization toward tumor-associated phenotypes.^18^

The biological effects of ADEs are further modulated by the anatomical origin of the adipose depot. In mammals, AT is classified into white (WAT), brown (BAT), and beige subtypes, with WAT being the most abundant and further subdivided into visceral (vWAT) and subcutaneous (sWAT) compartments. BAT and beige fat are primarily involved in non-shivering thermogenesis, whereas WAT functions predominantly as an energy reservoir.^19^ BAT-derived exosomes carry miRNAs such as miR-99b and miR-132-3p, which regulate hepatic FGF21 expression and lipid metabolism-related gene transcription, indicating a potential role in systemic metabolic regulation.^20,21^ By contrast, exosomes originating from vWAT – particularly under obese conditions – contain elevated levels of pro-inflammatory and tumor-associated mediators, including IL-6, macrophage migration inhibitory factor, TNF-α, and monocyte chemoattractant protein-1.^22,23^ These exosomes also exhibit the downregulation of tumor-suppressive miRNAs, such as miR-148b and miR-4269, alongside increased expression of the oncomiR miR-23b. Such molecular alterations have been specifically associated with breast cancer progression in the context of central obesity.^24^ Compared with exosomes derived from sWAT, those from vWAT are predicted to exert a greater influence on oncogenic signaling pathways, including the TGF-β and Wnt/β-catenin cascades, potentially contributing to stromal remodeling, immune evasion, and enhanced tumor cell proliferation.^25,26^

Obesity, defined by the World Health Organization as excessive fat accumulation, leads to systemic metabolic dysregulation and ectopic lipid redistribution beyond adipocytes.^27^ This pathological condition induces metabolic dysfunction, chronic inflammation, and immune alterations, collectively impairing DNA repair mechanisms, accelerating somatic mutations, and promoting epigenetic dysregulation – ultimately facilitating malignant transformation and cancer progression.^28,29^ In individuals with obesity, AT not only undergoes significant expansion in volume but also demonstrates a marked increase in exosome production compared with lean counterparts.^30,31^ Increasing evidence highlights AT, particularly through adipocyte-derived exosomes, as a key contributor to tumor progression, with these EVs emerging as central mediators in obesity-related oncogenesis.^32^

This review aims to provide a comprehensive overview of ADEs in cancer pathogenesis, with particular emphasis on their dual roles as both tumor promoters and suppressors in a context-dependent manner. By synthesizing current evidence across diverse molecular cargo types – including miRNAs, proteins, lipids, and long or circular RNAs – we outline how ADEs influence cancer progression through direct modulation of tumor cells, remodeling of the tumor microenvironment (TME), and activation of oncogenic signaling pathways. Moreover, we critically assess the translational potential of ADEs as diagnostic biomarkers, therapeutic targets, and bioengineered delivery vehicles, while identifying key barriers to clinical application. These include challenges related to standardization, biosafety, large-scale manufacturing, and, notably, the absence of clearly defined regulatory frameworks governing the development, quality control, and approval of exosome-based therapeutics. Collectively, this review aims to bridge critical gaps in our understanding of adipocyte – tumor communication and to inform the rational development of ADE-based strategies in precision oncology.

## 2. Biogenesis and characterization of ADEs

EVs constitute a heterogeneous population of membrane-bound nanocarriers that play essential roles in intercellular communication. Their secretion dynamics reflect the integrated physiological and pathological states of their cells of origin. EVs are operationally classified into three principal categories based on biogenetic origin and dimensional characteristics: exosomes (30–150 nm in diameter), which are derived from the endosomal system through multivesicular body (MVB) fusion with the plasma membrane; microvesicles (100‒1,000 nm), which are generated via calcium-dependent outward budding of the plasma membrane; and apoptotic bodies (1‒5 μm), which are formed during the terminal phase of programmed cell death as membrane-delimited carriers of cellular components.^33,34^ The historical trajectory of EV research can be traced back to a serendipitous observation in 1981 by Trams et al., who reported the release of membrane-associated enzymes accompanied by vesicular structures in tumor cell cultures – a phenomenon later identified as exosome secretion.^35^ This discovery was further substantiated in 1987 by Johnstone et al., who systematically characterized 50-nm vesicles released during reticulocyte maturation. This led to the formal designation of “exosomes” and laid the foundation for subsequent studies on EV biology.^36^

**Figure 1.**
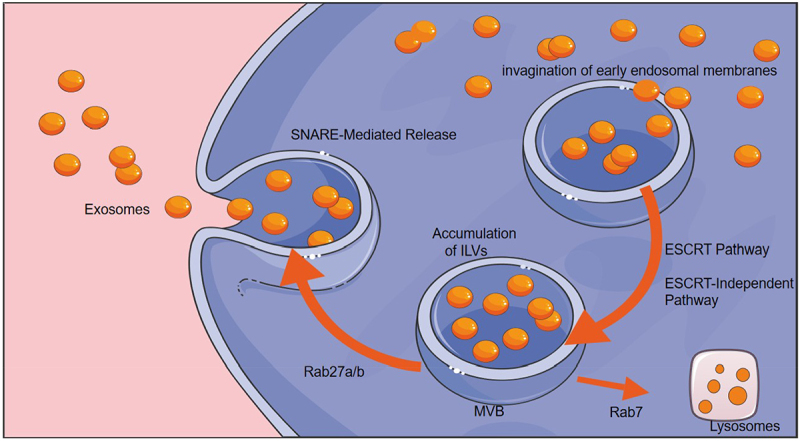
Schematic representation of exosome biogenesis showing key molecular pathways involved in exosome formation, including the ESCRT-dependent and ESCRT-independent mechanisms, Rab GTPase regulation, and SNARE-mediated release.

The biogenesis of exosomes is initiated by the progressive invagination of early endosomal membranes, ultimately resulting in the formation of intraluminal vesicles (ILVs) within maturing MVBs. This intricate process is coordinated through two principal mechanistic pathways: the canonical endosomal sorting complex required for transport (ESCRT) machinery and various ESCRT-independent mechanisms. In the ESCRT-dependent pathway, sequential recruitment of ESCRT-0 through ESCRT-III complexes facilitates the recognition of ubiquitinated cargo and membrane deformation. ESCRT-independent pathways include ceramide-mediated membrane microdomain formation driven by sphingomyelinase activity; syndecan-syntenin-apoptosis-linked-gene-2interacting-protein-X interactions, facilitating heparan sulfate-dependent cargo sorting; and the involvement of tetraspanin-enriched microdomains (including cluster of differentiation (CD) 63, CD81, and CD9) acting as spatial organizers for selective protein incorporation.^37,38^ During ILV maturation, selective loading of functional cargo, including miRNAs and metabolic enzymes, is completed prior to MVB formation. The fate of MVB is regulated by Rab GTPases: Rab7 directs lysosomal fusion for content degradation, whereas Rab27a/b guides MVBs to the plasma membrane for soluble N-ethylmaleimide-sensitive factor attachment protein receptors-mediated exosome release (Figure 1).^39,40^ Once released, exosomes are transported via the bloodstream to distant organs, where they interact with target cells through membrane fusion, ligand-receptor interactions, or internalization via phagocytosis.^41^

## 3. Roles of ADEs in cancer pathogenesis

### 3.1. Direct modulation of tumor cells by ADEs

A hallmark of primary human tumors is their unlimited replicative potential and inherent genomic instability.^42^ These features promote genetic heterogeneity across successive cell divisions, thereby enhancing tumor cell adaptability to microenvironmental cues through phenotypic plasticity.^43^ During this evolutionary process, subpopulations of tumor cells acquire malignant traits, including increased migratory and invasive capabilities, metastatic potential, tumor-initiating properties, and resistance to therapeutic agents. Notably, epithelial-to-mesenchymal transition (EMT) plays a central role in orchestrating these pleiotropic changes.^44^ EMT is a dynamic and reversible biological program in which epithelial cells lose their cell-cell adhesion and apical-basal polarity. This process drives their transition toward mesenchymal phenotypes by downregulating epithelial markers and acquiring mesenchymal markers. The resulting morphological transformation – from a cobblestone-like to a spindle-shaped structure – confers enhanced motility and invasive potential.^45^

Cancer stem cells (CSCs) are a subpopulation of tumor-initiating cells capable of self-renewal and differentiation into multiple cell lineages. They drive cancer development through two distinct division modes: symmetric division, which expands the CSC pool, and asymmetric division, which generates heterogeneous tumor cell progeny.^46^ The uncontrolled expansion of CSCs through sustained symmetric division is a key mechanism underlying tumor growth, progression, and recurrence.^47^

ADEs serve as pivotal mediators in these oncogenic processes by directly reprogramming tumor cell behavior through EMT induction and CSC activation. Kuhbier et al. demonstrated that co-culture with ADSCs promotes phenotypic transformation in breast cancer cells, enhancing their invasiveness. In this model, ADSCs formed monolayers around the tumor cells, facilitating direct cell-cell interactions and vesicle exchange.^48^ These findings suggest that ADSCs may contribute to tumor progression via ADE-mediated signaling. Similarly, ADEs upregulate genes associated with EMT and CSC traits in co-cultured breast cancer cells.^49^

Paradoxically, ADEs may also exert tumor-suppressive effects, depending on their cellular origin and the specific tumor context. Seo et al. demonstrated that miR-503-3p, an ADE-enriched miRNA, significantly inhibits CSC proliferation and self-renewal, thereby suppressing tumor growth. In MCF7 xenograft models, the intratumoral administration of miR-503-3p resulted in a 66% reduction in tumor volume.^50^ These findings highlight the dual regulatory role of ADEs in cancer progression and highlight the necessity of context-specific investigations to fully elucidate their functional implications.

### 3.2. Remodeling TME: ADEs as multifaceted regulators

**Figure 2.**
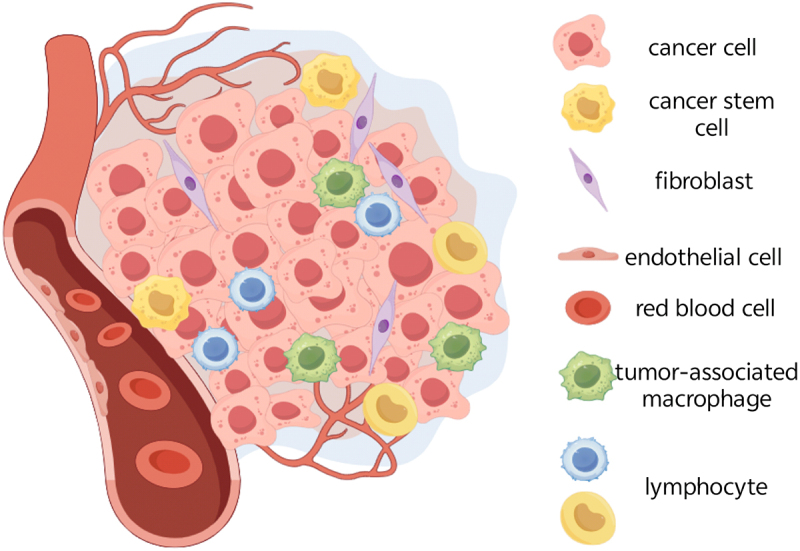
Schematic representation of the tumor microenvironment, illustrating various cellular components, including cancer cells, cancer stem cells, fibroblasts, endothelial cells, lymphocytes, and tumor-associated macrophages.

The TME is increasingly recognized as a critical determinant in the initiation, progression, and metastasis of cancer.^51^ This dynamic system maintains tissue homeostasis through coordinated interactions among malignant, endothelial, stromal, and immune cells (Figure 2).^52^ Although endothelial cells promote angiogenesis to support tumor growth and fibroblasts contribute to metastatic dissemination, immune cells represent the most functionally pivotal compartment within the TME.^53^ Among these, tumor-associated macrophages constitute the predominant immune population and exhibit remarkable plasticity in regulating various cancer hallmarks. They mediate immunosuppression primarily through M2 polarization (characterized by a CD206+/arginase-1 (Arg-1)+ phenotype), thereby protecting tumor cells from CD8+ T-cell-mediated cytotoxicity. Furthermore, tumor-associated macrophages facilitate the establishment of premetastatic niches by guiding circulating tumor cells to distant organs.^54,55^

ADEs are key modulators of TME remodeling, influencing immune cell polarization, stromal interactions, and extracellular matrix dynamics. Zhu et al. initially demonstrated that inflammatory-stimulated ADSC-exosomes (ADSC-Exos) promote tumor progression by inducing M2 macrophage polarization in THP-1 monocytes. This polarization is characterized by the upregulation of CD206 and Arg-1, downregulation of TNF-α and inducible nitric oxide synthase, and a reduction in CD3+/CD8+ T-cell ratio – collectively contributing to an immunosuppressive TME.^56^ In a subsequent study, these exosomes enhance cancer cell malignancy, including increased migration, proliferation, invasion, and apoptosis resistance, while concurrently suppressing M1 polarization in CD14+ monocytes.^57^

By contrast, findings by Ko et al. in hepatocellular carcinoma models revealed that ADEs may enhance natural killer T cell-mediated antitumor immunity, thereby suppressing hepatocellular carcinoma (HCC) progression.^58^ These paradoxical findings collectively highlight the context-dependent duality of ADEs in TME modulation, where their immunomodulatory effects and oncogenic potential vary based on cellular origin, stimulation conditions, and specific tumor milieus.

The immunosuppressive and pro-tumorigenic properties of ADEs are further amplified in obesity-associated TMEs. Obesity not only leads to AT expansion but also triggers chronic inflammation, characterized by adipocyte hypertrophy, immune cell infiltration, and inflammasome activation.^59^ This pathological state promotes a chronic inflammatory condition that actively supports cancer initiation and progression.^60^ Liu et al. demonstrated that obesity-driven ADEs facilitate ovarian cancer metastasis by enhancing lipogenesis, promoting vascularization, and suppressing M1 macrophage activation – further illustrating the complex interplay between metabolic dysregulation and cancer progression.^61^

### 3.3. Signaling network orchestration by ADEs

Exosomal regulation of oncogenic signaling pathways exhibits remarkable plasticity and context dependency. Lin et al. demonstrated that ADEs promote migratory behavior in MCF7 breast cancer cells by activating the Wnt pathway and upregulating migration-related genes.^62^ Notably, similar promigratory effects have been observed in other malignancies. Qu et al. reported that ADEs drive metastatic progression in epithelial ovarian carcinoma by enhancing FOXM1 signaling, thereby increasing tumor cell motility, invasive potential, and proliferative activity. Furthermore, ADEs were shown to facilitate the establishment of pro-metastatic niches via activation of the CXCL12/CXCR4 chemotaxis axis, contributing to peritoneal dissemination.^63^

The Hippo-YAP/TAZ signaling pathway has also been identified as a critical axis modulated by ADE. Wang et al. revealed that ADEs induce the nuclear translocation of YAP/TAZ transcriptional coactivators, leading to the upregulation of downstream targets – including CTGF, ANKDR1, and CYR61—which promote breast cancer cell proliferation and migration.^64^ This evidence suggests a spatially coordinated crosstalk among exosomal signaling modules, particularly within hormone-responsive cancers.

**Figure 3.**
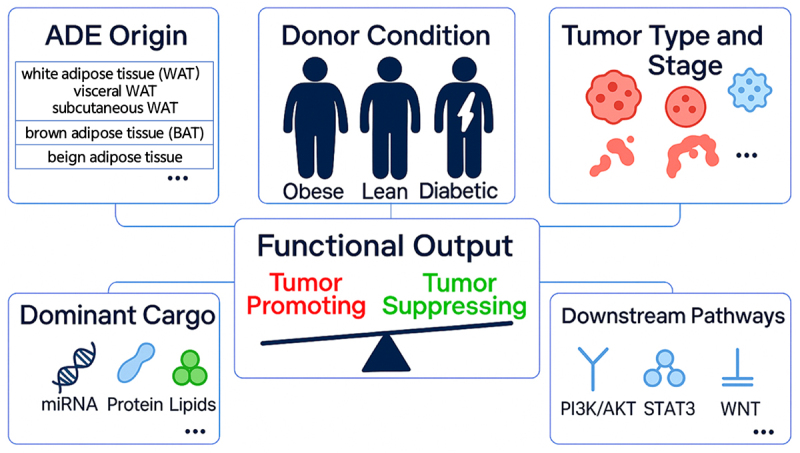
Key determinants of ADE functional variability in cancer. This schematic illustrates the principal biological parameters that collectively modulate the tumor-modulating roles of adipose-derived exosomes, including the anatomical origin of adipose depots, the metabolic condition of the donor, tumor characteristics, exosomal molecular cargo, and the signaling pathways activated in recipient cells. These interdependent factors determine whether ADEs exert tumor-promoting or tumor-suppressive effects within a given oncological context.

In summary, ADEs play multifaceted roles in cancer pathogenesis by reprogramming tumor cell behavior, remodeling the TME, and coordinating key oncogenic signaling pathways. These functional effects are not uniform, but rather depend on a constellation of interrelated biological variables, as illustrated in Figure 3. Such factors include the anatomical origin of adipose tissue (e.g., visceral, subcutaneous, white, brown, or beige fat), the donor’s metabolic status (e.g., obesity or diabetes), the molecular composition of exosomal cargo (including miRNAs, proteins, and lipids), as well as tumor-intrinsic features and the downstream signaling cascades they activate (e.g., PI3K/AKT, STAT3, and Wnt pathways).^65,66^ Together, these elements shape whether ADEs exert tumor-promoting or tumor-suppressive effects. This multifactorial regulation underscores the complexity of ADE-mediated interactions and highlights the need for deeper mechanistic investigations across diverse cancer contexts. Such understanding will be essential for harnessing or engineering ADEs for potential clinical applications, as further explored in the following sections.

## 4. Mechanistic insights: how ADEs modulate cancer cell behavior

### 4.1. miRNA in ADEs

MiRNAs are a class of single-stranded noncoding RNAs (typically 18‒28 nucleotides in length) that post-transcriptionally regulate gene expression through partial complementary binding to the 3′ untranslated regions (3′UTRs) of target messenger RNAs (mRNAs).^67,68^ This interaction, mediated by a conserved 6‒8 nucleotide seed sequence, facilitates either mRNA degradation or translational repression via the recruitment of the RNA-induced silencing complex.^69^ In addition to their intracellular regulatory functions, miRNAs are actively secreted within EVs – particularly from AT – functioning as endocrine signaling molecules.^70^ Increasing evidence has established the critical involvement of miRNAs in tumor pathogenesis, with dysregulated expression observed across nearly all human malignancies. These pathological alterations in miRNA expression profiles serve as key mediators of multistep carcinogenesis by modulating oncogenic signaling pathways and influencing processes such as malignant transformation, tumor progression, and metastatic dissemination.^71^

Hong Ji et al. identified three distinct EV subtypes (small microvesicles, A33-exosomes, and epithelial cell adhesion molecule-exosomes) – released by LIM1863 colon cancer cells, each exhibiting unique miRNA enrichment profiles. Among the 254 detected miRNAs, 63 showed significant enrichment in EVs. Kyoto Encyclopedia of Genes and Genomes pathway enrichment analysis of this 63-miRNA dataset revealed that the EV-derived miRNAs may regulate genes involved in critical signaling networks of recipient cells, including oncogenic pathways such as Wnt signaling, Ras signaling, transforming growth factor-beta signaling, and p53-mediated regulatory cascades.^72^ Notably, miRNAs contained within ADEs demonstrate dual regulatory capacities, not only facilitating pro-tumorigenic signaling but also exerting tumor-suppressive functions.

#### 4.1.1. Pro-tumorigenic miRnas in ADEs

In ovarian cancer models, ADEs deliver miR-421, which directly targets the chromatin modifier CBX7, leading to genome-wide histone modification changes that promote EMT and tumor progression.^73^ In HCC studies, ADEs act as principal transport carriers of miR-23a/b, which significantly enhance oncogenic behaviors, including cellular proliferation and invasive migration. Meanwhile, these exosomal miR-23a/b isoforms contribute to multidrug resistance by epigenetically silencing the von Hippel‒Lindau tumor suppressor. This silencing leads to the stabilization of hypoxia-inducible factor-1 alpha and the subsequent activation of chemoresistance pathways.^74^ While oncogenic miRNAs delivered via ADEs have been extensively characterized for their roles in promoting tumor progression, accumulating evidence also underscores the pivotal contribution of tumor-suppressive miRNAs within ADE cargo. Unlike their pro-tumorigenic counterparts, these miRNAs exhibit context-specific antitumor activity by modulating apoptosis, inhibiting metastasis, and restoring dysregulated signaling cascades. Notably, several of these suppressive miRNAs have demonstrated significant therapeutic potential, positioning ADEs as promising vehicles for miRNA-based interventions in cancer treatment.

#### 4.1.2. Tumor-suppressive miRnas in ADEs

The tumor-suppressive potential of EV-miRNAs is equally significant. Reza et al. reported that exosomes isolated from human adipose mesenchymal stem cell-conditioned medium significantly suppressed the viability, wound healing capacity, and colony formation of A2780 and SKOV-39 ovarian cancer cells. Notably, these inhibitory effects were retained following protease treatment that preserved vesicle integrity, whereas RNase digestion abolished the tumor-suppressive activity – confirming that the effects were mediated by miRNA-dependent mechanisms.^75^

Building on these findings, another study using ovarian cancer models demonstrated that ADEs markedly suppressed skin cancer cell proliferation and invasion by delivering miR-199a-5p. Mechanistically, miR-199a-5p directly targeted and downregulated SOX4 expression, as validated by luciferase reporter assays. SOX4, a transcription factor frequently overexpressed in malignancies, promotes tumor cell proliferation, migration, and EMT.^76^ By inhibiting SOX4, ADE-derived miR-199a-5p reduced oncogenic signaling and enhanced apoptosis in skin cancer cells, thereby exerting a potent tumor-suppressive effect.^77^

ADSC-derived miR-145 further exemplifies the therapeutic potential of ADE-associated microRNAs, inducing caspase-3/7 activation in PC3M-luc2 prostate cancer cells through direct targeting of apoptosis inhibitors. Notably, knockdown of miR-145 resulted in complete reversal of these pro-apoptotic effects, confirming its essential role in promoting apoptosis.^78^ Similarly, Lou et al. reported that the loss or downregulation of miR-122 is closely associated with HCC development and progression and is strongly correlated with poor prognosis and increased metastatic potential.^79,80^

In conclusion, miRNAs represent one of the most functionally diverse components of ADE cargo, exerting both oncogenic and tumor-suppressive effects in a context-dependent manner. A summary of key ADE-associated miRNAs and their respective roles in cancer is presented in Table 1. Their ability to regulate key signaling pathways, modulate the TME, and influence treatment response underscores their therapeutic relevance. Targeting oncogenic miRNAs or restoring tumor-suppressive miRNAs via exosome-mediated delivery may offer new opportunities for modulating cancer progression. Further elucidation of the mechanisms by which ADE-derived miRNAs influence tumor biology will be critical for translating these insights into clinically viable interventions

**Table 1.** Summary of miRnas in ADEs and their roles in cancer progression.

miRNA type	Effect direction	Mechanism/target	Associated cancer types	References
miR-503-3p	Anti-tumorigenic	Inhibits CSC proliferation/self-renewal	Breast cancer	^50^
miR-421	Pro-tumorigenic	Targets CBX7 to induce epigenetic modifications	Ovarian cancer	^73^
miR-23a/b	Pro-tumorigenic	Silences VHL to stabilize HIF-1α; induces chemoresistance	HCC	^74^
miR-199a-5p	Anti-tumorigenic	Suppresses SOX4 pathway; induces apoptosis	Skin cancer	^77^
miR-145	Anti-tumorigenic	Activates caspase-3/7 by targeting apoptosis inhibitors	Prostate cancer	^78^
miR-122	Anti-tumorigenic	Downregulated in HCC; associated with metastasis	HCC	^79,80^
miR-660-5p	Pro-tumorigenic	Enhances radioresistance via apoptosis/DNA damage suppression	Esophageal squamous cell carcinoma	^81^
miR-27a-3p	Anti-tumorigenic	Regulates ICOS+ T-cell activity; enhances immunotherapy response	Lung adenocarcinoma	^82^
miR-199a-3p	Pro-tumorigenic	Inhibits mTOR pathway; enhances chemosensitivity	HCC	^83^
miR-381	Anti-tumorigenic	Suppresses Wnt signaling (LRP6/CTNNB1); inhibits EMT	TNBC	^84^
miR-138-5p	Anti-tumorigenic	Targets survivin to inhibit migration, invasion, and proliferation	Bladder cancer	^85^

### 4.2. ADE cargo beyond miRnas: functional interplay of proteins, lipids, and long/circular RNAs

Beyond miRNAs, ADEs contain a diverse array of bioactive molecules – including proteins, lipids, circular RNAs (circRNAs), and long non-coding RNAs (lncRNAs) – that critically mediate tumor – stroma communication.

The interplay between exosomal proteins and lipids has emerged as a key mechanism underlying tumor progression by influencing both metabolic reprogramming and cellular signaling in the TME. The obesity-induced hypoxic microenvironment in AT significantly alters the composition of ADEs. Under such conditions, adipocytes exhibit increased exosomal protein expression associated with metabolic pathways.^86^ These altered ADEs enter systemic circulation, infiltrate the tumor stroma, and contribute to cancer-associated adipocyte maturation. In doing so, they provide energy substrates and biosynthetic precursors that fuel tumor growth and progression.^87^ For example, adipocyte-derived exosomes deliver fatty acids and fatty-acid oxidation (FAO) enzymes to melanoma cells, resulting in markedly increased FAO activity and enhanced tumor cell migration and invasion. Furthermore, Zhang et al. demonstrated that ADEs modulate both lipid metabolism and ferroptosis through the MTTP/PRAP1/ZEB1 signaling axis. Specifically, these vesicles suppress key lipid peroxidation enzymes while downregulating ferroptosis marker proteins, ultimately reducing chemotherapeutic sensitivity in colorectal cancer cells.^88^ This form of metabolic reprogramming contributes to the establishment of a protective niche that facilitates tumor adaptation to therapeutic stress.

Beyond their protein and lipid content, the non-miRNA RNA cargo of ADEs – particularly lncRNAs and circRNAs, further contributes to ADE-mediated tumor regulation.^89^ Wang et al. reported that bone marrow adipocyte-derived exosomes from individuals with obesity carry lncRNAs such as LOC606724 and SNHG1, which protect multiple myeloma cells from chemotherapy-induced apoptosis. These exosomal lncRNAs are upregulated in myeloma cells following ADE exposure and are correlated with poor clinical outcomes, highlighting their involvement in mediating chemoresistance.^90^ In triple-negative breast cancer (TNBC), Li et al. demonstrated that ADEs promote tumor progression through circCRIM1-mediated inhibition of miR-503-5p. Mechanistically, miR-503-5p directly targets the 3′UTR of OGA, thereby suppressing its expression and enzymatic activity. OGA, a key regulator of O-GlcNAcylation, promotes tumor growth by destabilizing fructose-1,6-bisphosphatase 1 (FBP1), a metabolic enzyme known for its tumor-suppressive properties.^91,92^ Through the downregulation of miR-503-5p, ADE-derived circCRIM1 facilitates OGA upregulation, leading to decreased FBP1 stability and promoting increased tumor cell glycolysis, proliferation, and metastatic potential in TNBC cells.^93^

In addition to miRNAs, ADEs carry a wide array of bioactive molecules, including proteins, lipids, lncRNAs, and circRNAs, each of which contributes significantly to tumor progression. These non-miRNA cargos modulate tumor metabolism, promote therapeutic resistance, and shape the TME by supporting cancer cell survival and adaptation under stress conditions. Several of these molecules, such as fatty acid oxidation enzymes, ferroptosis regulators, and lncRNAs associated with chemoresistance, have emerged as potential therapeutic targets. Elucidating the mechanisms through which these components exert their effects, and exploring strategies to selectively modify ADE cargo composition, may offer novel opportunities for developing exosome-based approaches to disrupt tumor-promoting signals and improve treatment outcomes.

## 5. Clinical implications of ADEs in oncology

Emerging preclinical evidence suggests the potential feasibility of ADE-based strategies in cancer intervention, although clinical translatability remains to be fully validated. García-Contreras et al. reported no significant differences in the miRNA profiles (such as let-7a-1, miR-21, miR-145, miR-451a, and miR-1908) between ADEs derived from patients with cancer and those from healthy donors. Moreover, molecular karyotyping of expanded ADSCs from patients with cancer further confirmed the absence of malignancy-associated genomic abnormalities, highlighting their therapeutic safety profile.^94^ Collectively, these findings support the potential of ADEs as promising candidates for clinical applications in oncology.

### 5.1. Ades as diagnostic biomarkers

The capacity of exosomes to transport tumor-associated biomolecules positions them as potential noninvasive biomarkers for cancer detection and treatment monitoring.^95^ Among these, ADEs play a particularly important role owing to their involvement in metabolic crosstalk within the TME, which directly influences therapeutic efficacy.^96^ Recent studies in esophageal squamous cell carcinoma (ESCC) have demonstrated that ADEs confer radiation resistance by protecting cancer cells from irradiation-induced apoptosis and DNA damage. This radioresistant phenotype correlates with specific miRNA signatures transported by ADEs, particularly miR-660-5p, which is consistently enriched in both AT and circulating exosomes of patients with ESCC. Clinical observations have identified serum exosomal miR-660-5p as a dynamic biomarker for evaluating therapeutic response. Its expression has been shown to inversely correlate with radiotherapy outcomes, with elevated baseline concentrations predicting poorer clinical response. Notably, successful treatment is associated with a measurable reduction in circulating miR-660-5p levels, underscoring the potential utility in real-time monitoring of treatment efficacy.^81^

### 5.2. Therapeutic targetability of ADEs

Beyond their role as diagnostic tools, ADEs exhibit significant therapeutic potential by modulating immune responses, drug sensitivity, and tumor progression. The identification of ADE-derived miR-27a-3p as a regulator of inducible T-cell co-stimulator (ICOS)+ T-cell activity provides mechanistic insight into the obesity paradox, wherein patients with obesity and lung adenocarcinoma (LUAD) demonstrate superior responses to immunotherapy.^97,98^ Clinical evidence has shown that plasma exosomal miR-27a-3p is significantly downregulated in patients with obesity and LUAD and correlates with ICOS expression. This reduction in ADE-derived miR-27a-3p relieves ICOS suppression, thereby enhancing T-cell proliferation and interferon-gamma secretion, positioning this molecular axis as a potentially tunable immune checkpoint.^82^ This form of endogenous exosome-mediated crosstalk is well-aligned with emerging strategies in therapeutic exosome engineering.

Similarly, ADSC-exos engineered to deliver miR-381 have demonstrated potent anti-tumor effects through dual-pathway modulation. These exosomes significantly inhibit Wnt signaling by downregulating LRP6 and CTNNB1, while simultaneously suppressing EMT progression through reduced expression of the transcription factors Twist and Snail. This dual inhibition reverses mesenchymal phenotypes, as demonstrated by Hojaei et al. Functionally, miR-381-enriched exosomes have been shown to reduce the proliferation, migration, and invasion of TNBC cells while promoting apoptosis in vitro.^84^

This combinatorial approach – simultaneously targeting oncogenic pathways and epigenetic reprogramming – highlights the distinct advantage of exosomes in overcoming tumor heterogeneity. Notably, these findings are consistent with prior successes involving miR-199a-enriched exosomes in HCC, further supporting the therapeutic relevance of this strategy. Collectively, ADSC-Exos have been established as versatile nanocarriers capable of delivering synergistic RNA therapeutics while preserving tumor-specific selectivity.

### 5.3. Exosome-mediated drug delivery

The advantages of ADEs in drug delivery vehicles arise from their inherent biocompatibility, structural stability, and low immunogenicity.^99,100^ Innovative strategies aimed at overcoming pharmacological limitations have been exemplified by thymoquinone (Tq) encapsulation. Although the anticancer efficacy of Tq is hindered by poor solubility and limited bioavailability, exosomal loading enhances its cytotoxic effects against MCF7 breast cancer cells while sparing normal L929 fibroblasts.^101^ Similarly, doxorubicin-loaded ADEs have demonstrated improved therapeutic indices compared with free drug formulations, achieving enhanced antitumor efficacy in breast cancer models while minimizing off-target toxicity.^102^

Researchers have successfully engineered miR-199a-3p-modified ADSCs via lentiviral transduction followed by puromycin selection, subsequently isolating miR-199a-enriched exosomes (ADSC-Exo-199a) from conditioned media. MiR-199a-3p, a microRNA abundantly expressed in normal hepatic tissue, is significantly downregulated in HCC, with its reduced expression strongly associated with poor clinical outcomes.^103,104^ These engineered exosomes retain classical morphological and molecular characteristics while efficiently delivering tumor-suppressive miR-199a-3p to HCC cells. Notably, ADSC-Exo-199a exerts dual therapeutic effects by restoring miR-199a-3p levels and simultaneously enhancing chemosensitivity through mTOR pathway inhibition.^83^ Similarly, Liu et al. used lentiviral transduction to generate ADSCs that stably expressed miR-138-5p. The resulting exosomes effectively delivered the miRNA to bladder cancer cells. These exosomes inhibited cell migration, invasion, and proliferation, reduced survivin expression, and significantly suppressed tumor growth in xenograft models through both local and systemic administration.^85^

This exosome-based delivery paradigm extends to nucleic acid therapeutics. In breast cancer *T*-47D cells, the therapeutic delivery of miR-145—either via direct administration or exosome-mediated transfer – was systematically evaluated. Both approaches were found to induce apoptosis and suppress metastasis. Crucially, the comparative analysis demonstrated that mesenchymal stem cell-derived exosomes (MSC-Exo) significantly enhanced the anticancer efficacy of miR-145 compared with naked miRNA delivery. This enhancement is likely attributable to improved cellular uptake and efficient endosomal escape mechanisms.^105^ These findings support MSC-Exo-mediated miR-145 restoration as a synergistic strategy that combines the benefits of nucleic acid with the inherent exosomal delivery, thus offering a promising translational approach for breast cancer management.

Collectively, these findings reinforce the potential of exosome-based drug delivery as a highly adaptable and effective strategy in cancer therapy. However, the standardization of manufacturing protocols, optimization of dosing regimens, and thorough assessment of potential immunogenic risks must be achieved before these exosome-based therapies can transition to clinical applications.

## 6. Current challenges and limitations

### 6.1. Heterogeneity and standardization

The clinical application of ADEs in cancer therapy is currently constrained by their inherent heterogeneity and the absence of standardized methodologies. Exosomes are secreted in diverse populations by cells, even within the same cell type, leading to variability in their molecular composition and targeting properties.^106^ This heterogeneity, influenced by factors such as the anatomical origin of AT, the metabolic condition of the donor, the molecular composition of the exosomal cargo and the TME, complicates the standardization of dosages, therapeutic delivery, and clinical applications. Recent studies employing single-vesicle imaging and proteomic profiling have revealed the presence of distinct exosomal subpopulations within individual biofluid samples, highlighting the intrinsic heterogeneity of exosomes and its adverse impact on experimental reproducibility.^107^

The Minimal Information for Studies of Extracellular Vesicles 2023 provides an updated framework for EV research, emphasizing the importance of standardized terminology, reproducibility, and transparent reporting practices. These guidelines are intended to improve the quality and consistency of EV-related studies, facilitating broader clinical and translational applicability.^108,109^

However, one of the principal challenges lies in the standardization of isolation and purification techniques. Commonly employed methods, such as ultracentrifugation and size-exclusion chromatography, often yield heterogeneous vesicle populations contaminated with non-exosomal components, thereby compromising reproducibility and hindering clinical validation. Emerging bioprocessing technologies, including tangential flow filtration and advanced chromatographic purification, could improve the yield and consistency of ADE production.^110^ Moreover, current Good Manufacturing Practice (cGMP) guidelines remain insufficiently defined for exosome-based therapeutics, complicating regulatory approval processes. Batch-to-batch variability, stability concerns, and lack of defined potency assays continue to hinder clinical translation. In parallel, artificial intelligence-powered analytics are being deployed to minimize operator-dependent variability and predict functional performance.^111^ Ultimately, overcoming the heterogeneity and standardization barriers will be a prerequisite for establishing ADEs as clinically viable exosome-based therapeutics. This will require sustained collaboration between researchers, industry stakeholders, and regulatory bodies to implement robust, reproducible, and scalable production pipelines.

### 6.2. Therapeutic efficacy and targeting

A critical obstacle in the advancement of ADE-based therapies is the assurance of clinical efficacy, particularly concerning efficient cargo loading and precise tumor targeting. To overcome limitations associated with natural exosome variability and limited drug-loading capacity, synthetic exosomes have been developed to mimic the structure and functionality of their natural counterparts. These bioengineered vesicles offer greater control over cargo loading, surface modifications, and targeting specificity. Synthetic exosomes can be modified chemically (e.g., click chemistry, PEGylation), genetically (e.g., fusion protein expression), or physically (e.g., electroporation, sonication) to enhance their therapeutic performance. Cargo can be loaded via endogenous methods – by transfecting donor cells prior to exosome production – or exogenously, using permeabilization techniques such as ultrasound, saponin treatment, freeze-thaw cycling, or electroporation. Each method exhibits variable encapsulation efficiency and structural integrity depending on the therapeutic agent being delivered.^112,113^

However, tumor-targeting specificity remains a significant challenge. Native ADEs often exhibit limited tumor-homing capabilities, resulting in off-target accumulation in organs such as the liver and spleen. Surface engineering approaches, including ligand conjugation and antibody modification, have demonstrated improved tumor selectivity in both *in vitro* systems and animal models. For example, folate-functionalized exosomes improve tumor retention and therapeutic efficacy compared with free drug administration.^114^ Similarly, the incorporation of cyclic RGD peptides (cRGD) into exosomes enhances their targeting capacity by specifically binding to αvβ3 integrin receptors, which are overexpressed on both tumor cells and tumor-associated endothelial cells. This modification substantially increases tumor retention, improves therapeutic efficacy, and reduces off-target effects, thereby presenting a promising strategy for more precise and effective cancer treatment.^115^ While these strategies hold significant promise, it is crucial to acknowledge that their efficacy has not yet been comprehensively evaluated in the context of ADEs, which are the primary focus of this study.

Another major challenge associated with exosome-based therapies is the difficulty in tracking exosomal biodistribution following administration. Due to their small size and capacity for uptake by various cells and tissues, the distribution of exosomes remains challenging to monitor, thereby complicating the assessment of their therapeutic efficacy. Modified ligands or antibodies may bind to unintended tissues, especially under systemic exposure, posing serious safety challenges. The development of reliable tracking methodologies is crucial for elucidating the mechanisms of action of exosomes and optimizing their clinical utility.^116^ Furthermore, *in vivo* validation is required to assess the translational potential of these engineered ADEs in human cancers. Each modification must be systematically evaluated to ensure that it effectively translates into improved anticancer efficacy *in vivo*.

### 6.3. Safety and immune considerations

The biological complexity of ADEs raises safety concerns, particularly regarding their capacity to transfer oncogenic miRNAs and metabolites. ADEs have been implicated in promoting tumor plasticity, facilitating immune evasion, and establishing pre-metastatic niches, necessitating comprehensive safety profiling. Although most studies focus on short-term efficacy, potential long-term risks such as tumor relapse and metastatic progression remain underexplored.

Immunogenicity also presents a notable concern. Although autologous ADEs are generally well-tolerated, the use of allogeneic or xenogeneic ADEs may elicit immune responses. Despite being considered less immunogenic than cell-based therapies, exosomes – particularly when administered repeatedly – may provoke complement activation or systemic inflammatory reactions, necessitating further preclinical immunogenicity assessments.

In addition, the source of AT raises ethical and safety concerns. Exosomes derived from elective liposuction procedures in healthy donors may differ significantly in cargo composition from those obtained during oncologic surgeries, introducing variability that may affect therapeutic consistency. The implementation of standardized donor screening protocols and tissue processing procedures is essential to mitigate these risks. Another critical limitation is the lack of intrinsic tumor selectivity in unmodified ADEs. When systemically administered, these exosomes may deliver bioactive cargo to non-target, healthy tissues, causing unintended effects. The application of tumor-targeting ligands or the incorporation of inducible safety switches in engineered ADEs may improve therapeutic precision while providing greater control over exosome activity.

### 6.4. Regulatory landscape and ethical considerations

Regulatory authorities in various countries and regions adopt different definitions and classifications for exosomes. In Europe, exosome therapeutics are classified as advanced therapeutic medicinal products; in the United States, no exosome-based cancer therapies – including those derived from ADEs – have received Food and Drug Administration approval. This regulatory gap highlights the importance of early engagement with regulatory agencies to ensure compliance with evolving guidelines. Key regulatory considerations include the establishment of standardized release criteria for ADE batches (such as particle size, zeta potential, surface marker expression, and cargo composition), along with assurance of batch-to-batch consistency and the conduct of toxicology studies in relevant animal models. Countries with underdeveloped regulations for exosome-based therapeutics should establish comprehensive legal frameworks to ensure safety, standardization, and clinical accountability in oncology applications.^117^

Achieving regulatory approval for ADE-based therapeutics will require strict adherence to rigorous standards typically applied to biologics, including well-defined manufacturing protocols, comprehensive preclinical evidence, and meticulously designed clinical trials that address safety endpoints. Beyond these foundational regulatory concerns, ethical considerations play a pivotal role in the responsible implementation of exosome-based therapies. Informed consent is critical, especially given that ADEs are often derived from patient biofluids or adipose tissues. Patients must be fully aware of how their biological materials will be collected, processed, and potentially reused, particularly in contexts involving genetic modification or long-term biobanking for future research. Transparency regarding the potential risks, benefits, and long-term implications of exosome-based interventions is essential to ensure ethical compliance and maintain public trust.^111^

## 7. Future directions and recommendations

To advance ADEs into clinical oncology, several critical priorities must be addressed. First, systematic studies are essential to understand the interactions between ADEs and tumor biology. Detailed molecular profiling of ADE-derived molecular cargo is essential to identify subpopulations that either promote or suppress tumor growth, guiding the selection or engineering of therapeutically relevant ADEs.

Second, bioengineering strategies must be optimized to enhance both the cargo loading and tumor-targeting capabilities. Surface modification – such as antibody conjugation or peptide ligand display – should be further optimized to improve their ability to selectively target tumor cells, potentially overcoming the current challenge of limited tumor-homing ability.^118^

The next critical priority is scalable biomanufacturing. Substantial investment is required in the development of bioreactor-based systems to support large-scale exosome production. In parallel, the standardization of purification methods, such as affinity capture and tangential flow filtration, must be pursued to ensure high purity and yield. Techniques like lyophilization should also be explored to extend shelf-life and enhance stability, making ADEs more viable for clinical use.^119^ To facilitate this, cGMP-compliant ADE production pipelines must be established through public-private partnerships, which will be essential for meeting regulatory requirements and enabling consistent, reproducible exosome production.

In parallel, regulatory and ethical guidelines governing ADE-based therapies must be adapted to keep pace with ongoing technological advancements. The development of dedicated regulatory guidelines for exosome-based therapies is essential to establish harmonized global standards for clinical implementation. This process should include the standardization of donor selection criteria and the formulation of transparent tissue sourcing protocols, thereby ensuring the safety and ethical compliance of ADE-based therapies.

In addition, the personalization of ADE-based therapies is crucial for maximizing clinical benefit. By integrating patient-specific factors, such as metabolic profile, TME, and genetic markers, into the ADE design, therapeutic interventions can be tailored to the individual needs of each patient. Combining ADEs with immune checkpoint inhibitors or targeted therapies may yield synergistic antitumor effects, thereby enhancing overall therapeutic efficacy.

## References

[1] Coelho M, Oliveira T, Fernandes R. Biochemistry of adipose tissue: an endocrine organ. Archiv Med Sci. 2013;9(2):191–19. doi: 10.5114/aoms.2013.33181. PMC3648822 23671428

[2] Gimble JM, Bunnell BA, Frazier T, Rowan B, Shah F, Thomas-Porch C, Wu X. Adipose-derived stromal/stem cells: a primer. Organogenesis. 2013;9(1):3–10. doi: 10.4161/org.24279. 23538753 PMC3674038

[3] Michel LYM. Extracellular vesicles in adipose tissue communication with the healthy and pathological heart. Int J Mol Sci. 2023;24(9):7745. doi: 10.3390/ijms24097745. 37175451 PMC10177965

[4] Psaraki A, Ntari L, Karakostas C, Korrou-Karava D, Roubelakis MG. Extracellular vesicles derived from mesenchymal stem/stromal cells: the regenerative impact in liver diseases. Hepatol (baltim, Md). 2022;75(6):1590–1603. doi: 10.1002/hep.32129. 34449901

[5] Tetta C, Ghigo E, Silengo L, Deregibus MC, Camussi G. Extracellular vesicles as an emerging mechanism of cell-to-cell communication. Endocrine. 2013;44(1):11–19. doi: 10.1007/s12020-012-9839-0. 23203002 PMC3726927

[6] Chen CY, Rao SS, Ren L, Hu XK, Tan YJ, Hu Y, Luo J, Liu YW, Yin H, Huang J, et al. Exosomal DMBT1 from human urine-derived stem cells facilitates diabetic wound repair by promoting angiogenesis. Theranostics. 2018;8(6):1607–1623. doi: 10.7150/thno.22958. 29556344 PMC5858170

[7] Li X, Ballantyne LL, Yu Y, Funk CD. Perivascular adipose tissue-derived extracellular vesicle miR-221-3p mediates vascular remodeling. FASEB J. 2019;33(11):12704–12722. doi: 10.1096/fj.201901548R. 31469602 PMC6902668

[8] Keshtkar S, Azarpira N, Ghahremani MH. Mesenchymal stem cell-derived extracellular vesicles: novel frontiers in regenerative medicine. STEM Cell Res Ther. 2018;9(1):63. doi: 10.1186/s13287-018-0791-7. 29523213 PMC5845209

[9] Ritter A, Kreis NN, Hoock SC, Solbach C, Louwen F, Yuan J. Adipose tissue-derived mesenchymal stromal/stem cells, obesity and the tumor microenvironment of breast cancer. Cancers. 2022;14(16):3908. doi: 10.3390/cancers14163908. 36010901 PMC9405791

[10] Goodarzi P, Larijani B, Alavi-Moghadam S, Tayanloo-Beik A, Mohamadi-Jahani F, Ranjbaran N, Payab M, Falahzadeh K, Mousavi M, Arjmand B. Mesenchymal stem cells-derived exosomes for wound regeneration. Adv Exp Med Biol. 2018;1119:119–131. 30051320 10.1007/5584_2018_251

[11] Kang Y, Xu C, Meng L, Dong X, Qi M, Jiang D. Exosome-functionalized magnesium-organic framework-based scaffolds with osteogenic, angiogenic and anti-inflammatory properties for accelerated bone regeneration. Bioact Mater. 2022;18:26–41. doi: 10.1016/j.bioactmat.2022.02.012. 35387167 PMC8961306

[12] Fatima F, Nawaz M. Long distance metabolic regulation through adipose-derived circulating exosomal miRnas: a trail for RNA-Based therapies? Frontiers in physiology. Front Physiol. 2017;8:545. doi: 10.3389/fphys.2017.00545. 28824444 PMC5539684

[13] Żbikowski A, Błachnio-Zabielska A, Galli M, Zabielski P. Adipose-derived exosomes as possible players in the development of insulin resistance. Int J Mol Sci. 2021;22(14):7427. doi: 10.3390/ijms22147427. 34299048 PMC8304687

[14] Wu Q, Li B, Li Z, Li J, Sun S, Sun S. Cancer-associated adipocytes: key players in breast cancer progression. J Hematol Oncol. 2019;12(1):95. doi: 10.1186/s13045-019-0778-6. 31500658 PMC6734503

[15] Afrin S, Ramaiyer M, Begum UAM, Borahay MA. Adipocyte and adipokines promote a uterine leiomyoma friendly microenvironment. Nutrients. 2023;15(3):715. doi: 10.3390/nu15030715. 36771423 PMC9919329

[16] Lin Z, Wu Y, Xu Y, Li G, Li Z, Liu T. Mesenchymal stem cell-derived exosomes in cancer therapy resistance: recent advances and therapeutic potential. Mol Cancer. 2022;21(1):179. doi: 10.1186/s12943-022-01650-5. 36100944 PMC9468526

[17] Wan FZ, Chen KH, Sun YC, Chen XC, Liang RB, Chen L, Zhu XD. Exosomes overexpressing miR-34c inhibit malignant behavior and reverse the radioresistance of nasopharyngeal carcinoma. J Transl Med. 2020;18(1):12. doi: 10.1186/s12967-019-02203-z. 31915008 PMC6947927

[18] Yoshida K, Yokoi A, Kato T, Ochiya T, Yamamoto Y. The clinical impact of intra- and extracellular miRnas in ovarian cancer. Cancer Sci. 2020;111(10):3435–3444. doi: 10.1111/cas.14599. 32750177 PMC7541008

[19] Dong K, Wei G, Sun H, Gu D, Liu J, Wang L. Metabolic crosstalk between thermogenic adipocyte and cancer cell: dysfunction and therapeutics. Curr Opin Pharmacol. 2023;68:102322. doi: 10.1016/j.coph.2022.102322. 36502545

[20] Thomou T, Mori MA, Dreyfuss JM, Konishi M, Sakaguchi M, Wolfrum C, Rao TN, Winnay JN, Garcia-Martin R, Grinspoon SK, et al. Adipose-derived circulating miRnas regulate gene expression in other tissues. Nature. 2017;542(7642):450–455. doi: 10.1038/nature21365. 28199304 PMC5330251

[21] Kariba Y, Yoshizawa T, Sato Y, Tsuyama T, Araki E, Yamagata K. Brown adipocyte-derived exosomal miR-132-3p suppress hepatic Srebf1 expression and thereby attenuate expression of lipogenic genes. Biochem Bioph Res Co. 2020;530(3):500–507. doi: 10.1016/j.bbrc.2020.05.090. 32595040

[22] Kranendonk ME, Visseren FL, van Herwaarden JA, Nolte-‘t Hoen EN, de Jager W, Wauben MH, Kalkhoven E. Effect of extracellular vesicles of human adipose tissue on insulin signaling in liver and muscle cells. Obes (silver Spring, Md). 2014;22(10):2216–2223. doi: 10.1002/oby.20847. 25045057

[23] Liu Y, Wang C, Wei M, Yang G, Yuan L. Multifaceted roles of adipose tissue-derived exosomes in physiological and pathological conditions. Front Physiol. 2021;12:669429. doi: 10.3389/fphys.2021.669429. 33959041 PMC8093393

[24] Zimta AA, Tigu AB, Muntean M, Cenariu D, Slaby O, Berindan-Neagoe I. Molecular links between central obesity and breast cancer. Int J Mol Sci. 2019;20(21):5364. doi: 10.3390/ijms20215364. 31661891 PMC6862548

[25] Ferrante SC, Nadler EP, Pillai DK, Hubal MJ, Wang Z, Wang JM, Gordish-Dressman H, Koeck E, Sevilla S, Wiles AA, et al. Adipocyte-derived exosomal miRnas: a novel mechanism for obesity-related disease. Pediatr Res. 2015;77(3):447–454. doi: 10.1038/pr.2014.202. 25518011 PMC4346410

[26] Gao X, Salomon C, Freeman DJ. Extracellular vesicles from adipose tissue—A potential role in obesity and type 2 diabetes? Front Endocrinol. 2017;8:202. doi: 10.3389/fendo.2017.00202. PMC5563356 28868048

[27] Avgerinos KI, Spyrou N, Mantzoros CS, Dalamaga M. Obesity and cancer risk: emerging biological mechanisms and perspectives. Metab. 2019;92:121–135. doi: 10.1016/j.metabol.2018.11.001. 30445141

[28] Dalamaga M, Christodoulatos GS, Mantzoros CS. The role of extracellular and intracellular nicotinamide phosphoribosyl-transferase in cancer: diagnostic and therapeutic perspectives and challenges. Metab. 2018;82:72–87. doi: 10.1016/j.metabol.2018.01.001. 29330025

[29] Dalamaga M, Diakopoulos KN, Mantzoros CS. The role of adiponectin in cancer: a review of current evidence. Endocr Rev. 2012;33(4):547–594. doi: 10.1210/er.2011-1015. 22547160 PMC3410224

[30] Kranendonk ME, Visseren FL, van Balkom BW, Nolte-‘t Hoen EN, van Herwaarden JA, de Jager W, Schipper HS, Brenkman AB, Verhaar MC, Wauben MH, et al. Human adipocyte extracellular vesicles in reciprocal signaling between adipocytes and macrophages. Obes (silver Spring, Md). 2014;22(5):1296–1308. doi: 10.1002/oby.20679. 24339422

[31] Lazar I, Clement E, Dauvillier S, Milhas D, Ducoux-Petit M, LeGonidec S, Moro C, Soldan V, Dalle S, Balor S, et al. Adipocyte exosomes promote melanoma aggressiveness through fatty acid oxidation: a novel mechanism linking obesity and cancer. Cancer Res. 2016;76(14):4051–4057. doi: 10.1158/0008-5472.CAN-16-0651. 27216185

[32] Clement E, Lazar I, Muller C, Nieto L. Obesity and melanoma: could fat be fueling malignancy? Pigment cell & melanoma research. Pigment Cell & Melanoma Res. 2017;30(3):294–306. doi: 10.1111/pcmr.12584. 28222242

[33] Kalluri R, LeBleu VS. The biology, function, and biomedical applications of exosomes. Sci (new Y, NY). 2020;367(6478):eaau6977. doi: 10.1126/science.aau6977. PMC7717626 32029601

[34] Sonbhadra S, Mehak M, Pandey LM. Biogenesis, isolation, and detection of exosomes and their potential in therapeutics and diagnostics. Biosens (Basel). 2023;13(8):802. doi: 10.3390/bios13080802. PMC10452587 37622888

[35] Trams EG, Lauter CJ, Salem N Jr., Heine U. Exfoliation of membrane ecto-enzymes in the form of micro-vesicles. Biochim Et Biophys Acta. 1981;645(1):63–70. doi: 10.1016/0005-2736(81)90512-5. 6266476

[36] Johnstone RM. Revisiting the road to the discovery of exosomes. Blood Cells Mol Dis. 2005;34(3):214–219. doi: 10.1016/j.bcmd.2005.03.002. 15885604

[37] Trajkovic K, Hsu C, Chiantia S, Rajendran L, Wenzel D, Wieland F, Schwille P, Brügger B, Simons M. Ceramide triggers budding of exosome vesicles into multivesicular endosomes. Science (New York, NY). 2008;319(5867):1244–1247. doi: 10.1126/science.1153124. 18309083

[38] van Niel G, Carter DRF, Clayton A, Lambert DW, Raposo G, Vader P. Challenges and directions in studying cell-cell communication by extracellular vesicles. Nat Rev Mol Cell Biol. 2022;23(5):369–382. doi: 10.1038/s41580-022-00460-3. 35260831

[39] Ostrowski M, Carmo NB, Krumeich S, Fanget I, Raposo G, Savina A, Moita CF, Schauer K, Hume AN, Freitas RP, et al. Rab27a and Rab27b control different steps of the exosome secretion pathway. Nat Cell Biol. 2010;12(1):19–30; sup pp 11–13. doi: 10.1038/ncb2000. 19966785

[40] Colombo M, Raposo G, Théry C. Théry C: biogenesis, secretion, and intercellular interactions of exosomes and other extracellular vesicles. Annu Rev Cell Dev Biol. 2014;30(1):255–289. doi: 10.1146/annurev-cellbio-101512-122326. 25288114

[41] Singh S, Paul D, Nath V, R A. Exosomes: current knowledge and future perspectives. Tissue Barriers. 2024;12(2):2232248. doi: 10.1080/21688370.2023.2232248. 37439246 PMC11042064

[42] Hanahan D, Weinberg RA. The hallmarks of cancer. Cell. 2000;100(1):57–70. doi: 10.1016/S0092-8674(00)81683-9. 10647931

[43] Marusyk A, Almendro V, Polyak K. Intra-tumour heterogeneity: a looking glass for cancer? Nat Rev Cancer. 2012;12(5):323–334. doi: 10.1038/nrc3261. 22513401

[44] Marcucci F, Bellone M, Caserta CA, Corti A. Pushing tumor cells towards a malignant phenotype: stimuli from the microenvironment, intercellular communications and alternative roads. Int J Cancer. 2014;135(6):1265–1276. doi: 10.1002/ijc.28572. 24174383

[45] Dongre A, Weinberg RA. New insights into the mechanisms of epithelial-mesenchymal transition and implications for cancer. Nat Rev Mol Cell Biol. 2019;20(2):69–84. doi: 10.1038/s41580-018-0080-4. 30459476

[46] Matsui W, Huff CA, Wang Q, Malehorn MT, Barber J, Tanhehco Y, Smith BD, Civin CI, Jones RJ. Characterization of clonogenic multiple myeloma cells. Blood. 2004;103(6):2332–2336. doi: 10.1182/blood-2003-09-3064. 14630803 PMC3311914

[47] Yang L, Shi P, Zhao G, Xu J, Peng W, Zhang J, Zhang G, Wang X, Dong Z, Chen F, et al. Targeting cancer stem cell pathways for cancer therapy. Sig Transduct Target Ther. 2020;5(1):8. doi: 10.1038/s41392-020-0110-5. PMC7005297 32296030

[48] Kuhbier JW, Bucan V, Reimers K, Strauss S, Lazaridis A, Jahn S, Radtke C, Vogt PM. Observed changes in the morphology and phenotype of breast cancer cells in direct co-culture with adipose-derived stem cells. Plast And Reconstructive Surg. 2014;134(3):414–423. doi: 10.1097/PRS.0000000000000525. 25158701

[49] Jafari N, Kolla M, Meshulam T, Shafran JS, Qiu Y, Casey AN, Pompa IR, Ennis CS, Mazzeo CS, Rabhi N, et al. Adipocyte-derived exosomes may promote breast cancer progression in type 2 diabetes. Sci Signal. 2021;14(710):eabj2807. doi: 10.1126/scisignal.abj2807. 34813359 PMC8765301

[50] Seo M, Kim SM, Woo EY, Han KC, Park EJ, Ko S, Choi EW, Jang M. Stemness-attenuating miR-503-3p as a paracrine factor to regulate growth of cancer stem cells. STEM Cells Int. 2018;2018:4851949. doi: 10.1155/2018/4851949. 29849663 PMC5904772

[51] Mbeunkui F, Johann DJ Jr. Cancer and the tumor microenvironment: a review of an essential relationship. Cancer Chemother Pharmacol. 2009;63(4):571–582. doi: 10.1007/s00280-008-0881-9. 19083000 PMC2858592

[52] Vitale I, Manic G, Coussens LM, Kroemer G, Galluzzi L. Macrophages and metabolism in the tumor microenvironment. Cell Metab. 2019;30(1):36–50. doi: 10.1016/j.cmet.2019.06.001. 31269428

[53] Arneth B. Tumor microenvironment. Medicina (kaunas, Lithuania). 2019;56(1):15. doi: 10.3390/medicina56010015. 31906017 PMC7023392

[54] Hanahan D, Weinberg RA. Hallmarks of cancer: the next generation. Cell. 2011;144(5):646–674. doi: 10.1016/j.cell.2011.02.013. 21376230

[55] Mantovani A, Allavena P, Sica A, Balkwill F. Cancer-related inflammation. Nature. 2008;454(7203):436–444. doi: 10.1038/nature07205. 18650914

[56] Zhu Q, Zhang K, Cao Y, Hu Y. Adipose stem cell exosomes, stimulated by pro-inflammatory factors, enhance immune evasion in triple-negative breast cancer by modulating the HDAC6/STAT3/PD-L1 pathway through the transporter UCHL1. Cancer Cell Int. 2024;24(1):385. doi: 10.1186/s12935-024-03557-1. 39568023 PMC11577656

[57] Zhu Q, Cao Y, Yuan J, Hu Y. Adipose-derived stem cell exosomes promote tumor characterization and immunosuppressive microenvironment in breast cancer. Cancer Immunol Immunother. 2024;73(2):39. doi: 10.1007/s00262-023-03584-3. 38294569 PMC10830720

[58] Ko SF, Yip HK, Zhen YY, Lee CC, Lee CC, Huang CC, Ng SH, Lin JW. Adipose-derived mesenchymal stem cell exosomes suppress hepatocellular carcinoma growth in a rat model: apparent diffusion coefficient, Natural killer T-Cell responses, and histopathological features. STEM Cells Int. 2015;2015:853506. doi: 10.1155/2015/853506. 26345219 PMC4545422

[59] Suganami T, Nishida J, Ogawa Y. A paracrine loop between adipocytes and macrophages aggravates inflammatory changes: role of free fatty acids and tumor necrosis factor alpha. Arterioscler Thromb Vasc Biol. 2005;25(10):2062–2068. doi: 10.1161/01.ATV.0000183883.72263.13. 16123319

[60] Deng T, Lyon CJ, Bergin S, Caligiuri MA, Hsueh WA. Obesity, inflammation, and cancer. Annu Rev Pathol Mech Dis. 2016;11(1):421–449. doi: 10.1146/annurev-pathol-012615-044359. 27193454

[61] Liu Y, Metzinger MN, Lewellen KA, Cripps SN, Carey KD, Harper EI, Shi Z, Tarwater L, Grisoli A, Lee E, et al. Obesity contributes to ovarian cancer metastatic success through increased lipogenesis, enhanced vascularity, and decreased infiltration of M1 macrophages. Cancer Res. 2015;75(23):5046–5057. doi: 10.1158/0008-5472.CAN-15-0706. 26573796 PMC4668203

[62] Lin R, Wang S, Zhao RC. Exosomes from human adipose-derived mesenchymal stem cells promote migration through Wnt signaling pathway in a breast cancer cell model. Mol Cell Biochem. 2013;383(1–2):13–20. doi: 10.1007/s11010-013-1746-z. 23812844

[63] Qu Q, Liu L, Cui Y, Chen Y, Wang Y, Wang Y. Exosomes from human omental adipose-derived mesenchymal stem cells secreted into ascites promote peritoneal metastasis of epithelial ovarian cancer. Cells. 2022;11(21):3392. doi: 10.3390/cells11213392. 36359787 PMC9655202

[64] Wang S, Su X, Xu M, Xiao X, Li X, Li H, Keating A, Zhao RC. Exosomes secreted by mesenchymal stromal/stem cell-derived adipocytes promote breast cancer cell growth via activation of Hippo signaling pathway. STEM Cell Res Ther. 2019;10(1):117. doi: 10.1186/s13287-019-1220-2. 30971292 PMC6458638

[65] Wang Y, Li Q, Zhou S, Tan P. Contents of exosomes derived from adipose tissue and their regulation on inflammation, tumors, and diabetes. Front Endocrinol. 2024;15:1374715. doi: 10.3389/fendo.2024.1374715. PMC11361949 39220365

[66] Quan M, Kuang S. Exosomal secretion of adipose tissue during various physiological states. Pharm Res. 2020;37(11):221. doi: 10.1007/s11095-020-02941-6. 33063193 PMC7953939

[67] Bartel DP. MicroRNAs: genomics, biogenesis, mechanism, and function. Cell. 2004;116(2):281–297. doi: 10.1016/S0092-8674(04)00045-5. 14744438

[68] He L, Hannon GJ. MicroRNAs: small RNAs with a big role in gene regulation. Nat Rev Genet. 2004;5(7):522–531. doi: 10.1038/nrg1379. 15211354

[69] Ameres SL, Martinez J, Schroeder R. Molecular basis for target RNA recognition and cleavage by human RISC. Cell. 2007;130(1):101–112. doi: 10.1016/j.cell.2007.04.037. 17632058

[70] Hong P, Yu M, Tian W. Diverse RNAs in adipose-derived extracellular vesicles and their therapeutic potential. Mol Ther Nucleic Acids. 2021;26:665–677. doi: 10.1016/j.omtn.2021.08.028. 34703651 PMC8516999

[71] Adams BD, Kasinski AL, Slack FJ. Aberrant regulation and function of microRNAs in cancer. Curr Biol. 2014;24(16):R762–R776. doi: 10.1016/j.cub.2014.06.043. 25137592 PMC4177046

[72] Ji H, Chen M, Greening DW, He W, Rai A, Zhang W, Simpson RJ. Deep sequencing of RNA from three different extracellular vesicle (EV) subtypes released from the human LIM1863 colon cancer cell line uncovers distinct miRNA-enrichment signatures. PLOS ONE. 2014;9(10):e110314. doi: 10.1371/journal.pone.0110314. 25330373 PMC4201526

[73] Zhang Y, Tedja R, Millman M, Wong T, Fox A, Chehade H, Gershater M, Adzibolosu N, Gogoi R, Anderson M, et al. Adipose-derived exosomal miR-421 targets CBX7 and promotes metastatic potential in ovarian cancer cells. J Ovarian Res. 2023;16(1):233. doi: 10.1186/s13048-023-01312-0. 38037081 PMC10688490

[74] Liu Y, Tan J, Ou S, Chen J, Chen L. Adipose-derived exosomes deliver miR-23a/b to regulate tumor growth in hepatocellular cancer by targeting the VHL/HIF axis. J Physiol Biochem. 2019;75(3):391–401. doi: 10.1007/s13105-019-00692-6. 31321740

[75] Reza A, Choi YJ, Yasuda H, Kim JH. Human adipose mesenchymal stem cell-derived exosomal-miRnas are critical factors for inducing anti-proliferation signalling to A2780 and SKOV-3 ovarian cancer cells. Sci Rep. 2016;6(1):38498. doi: 10.1038/srep38498. 27929108 PMC5143979

[76] Chen J, Ju HL, Yuan XY, Wang TJ, Lai BQ. SOX4 is a potential prognostic factor in human cancers: a systematic review and meta-analysis. Clin Transl Oncol. 2016;18(1):65–72. doi: 10.1007/s12094-015-1337-4. 26250764

[77] Liu M, Wang H, Liu Z, Liu G, Wang W, Li X. Exosomes from adipose-derived stem cells inhibits skin cancer progression via miR-199a-5p/SOX4. Biotechnol Genet Eng Rev. 2024;40(4):3950–3962. doi: 10.1080/02648725.2023.2204702. 37092869

[78] Takahara K, Ii M, Inamoto T, Nakagawa T, Ibuki N, Yoshikawa Y, Tsujino T, Uchimoto T, Saito K, Takai T, et al. microRNA-145 mediates the inhibitory effect of adipose tissue-derived stromal cells on prostate cancer. STEM Cells Devel. 2016;25(17):1290–1298. doi: 10.1089/scd.2016.0093. 27465939

[79] Coulouarn C, Factor VM, Andersen JB, Durkin ME, Thorgeirsson SS. Loss of miR-122 expression in liver cancer correlates with suppression of the hepatic phenotype and gain of metastatic properties. Oncogene. 2009;28(40):3526–3536. doi: 10.1038/onc.2009.211. 19617899 PMC3492882

[80] Lou G, Song X, Yang F, Wu S, Wang J, Chen Z, Liu Y. Exosomes derived from miR-122-modified adipose tissue-derived MSCs increase chemosensitivity of hepatocellular carcinoma. J Hematol Oncol. 2015;8(1):122. doi: 10.1186/s13045-015-0220-7. 26514126 PMC4627430

[81] Ge YY, Xia XC, Wu AQ, Ma CY, Yu LH, Zhou JY. Identifying adipocyte-derived exosomal miRnas as potential novel prognostic markers for radiotherapy of esophageal squamous cell carcinoma. World J Gastrointest Oncol. 2025;17(2):98808. doi: 10.4251/wjgo.v17.i2.98808. 39958561 PMC11756016

[82] Fan X, Wang J, Qin T, Zhang Y, Liu W, Jiang K, Huang D. Exosome miR-27a-3p secreted from adipocytes targets ICOS to promote antitumor immunity in lung adenocarcinoma. Thorac Cancer. 2020;11(6):1453–1464. doi: 10.1111/1759-7714.13411. 32212417 PMC7262893

[83] Lou G, Chen L, Xia C, Wang W, Qi J, Li A, Zhao L, Chen Z, Zheng M, Liu Y. MiR-199a-modified exosomes from adipose tissue-derived mesenchymal stem cells improve hepatocellular carcinoma chemosensitivity through mTOR pathway. J Exp Clin Cancer Res. 2020;39(1):4. doi: 10.1186/s13046-019-1512-5. 31898515 PMC6941283

[84] Shojaei S, Hashemi SM, Ghanbarian H, Sharifi K, Salehi M, Mohammadi-Yeganeh S. Delivery of miR-381-3p mimic by mesenchymal stem cell-derived exosomes inhibits triple negative breast cancer Aggressiveness; an in vitro study. STEM Cell Rev And Rep. 2021;17(3):1027–1038. doi: 10.1007/s12015-020-10089-4. 33410095

[85] Liu T, Li T, Zheng Y, Xu X, Sun R, Zhan S, Guo X, Zhao Z, Zhu W, Feng B, et al. Evaluating adipose-derived stem cell exosomes as miRNA drug delivery systems for the treatment of bladder cancer. Cancer Med. 2022;11(19):3687–3699. doi: 10.1002/cam4.4745. 35441482 PMC9554444

[86] Sano S, Izumi Y, Yamaguchi T, Yamazaki T, Tanaka M, Shiota M, Osada-Oka M, Nakamura Y, Wei M, Wanibuchi H, et al. Lipid synthesis is promoted by hypoxic adipocyte-derived exosomes in 3T3-L1 cells. Biochem Bioph Res Co. 2014;445(2):327–333. doi: 10.1016/j.bbrc.2014.01.183. 24513287

[87] Feng S, Lou K, Luo C, Zou J, Zou X, Zhang G. Obesity-related cross-talk between prostate cancer and peripheral fat: potential role of exosomes. Cancers. 2022;14(20):5077. doi: 10.3390/cancers14205077. 36291860 PMC9600017

[88] Zhang Q, Deng T, Zhang H, Zuo D, Zhu Q, Bai M, Liu R, Ning T, Zhang L, Yu Z, et al. Adipocyte-derived exosomal MTTP suppresses ferroptosis and promotes chemoresistance in colorectal cancer. Adv Sci (weinheim, baden-Wurttemberg, Germany). 2022;9(28):e2203357. doi: 10.1002/advs.202203357. PMC9534973 35978266

[89] Aakel N, Mohammed R, Fathima A, Kerzabi R, Abdallah A, Ibrahim WN. Role of exosome in solid cancer progression and its potential therapeutics in cancer treatment. Cancer Med. 2025;14(9):e70941. doi: 10.1002/cam4.70941. 40344389 PMC12063069

[90] Wang Z, He J, Bach DH, Huang YH, Li Z, Liu H, Lin P, Yang J. Induction of m(6)A methylation in adipocyte exosomal LncRNAs mediates myeloma drug resistance. J Exp Clin Cancer Res. 2022;41(1):4. doi: 10.1186/s13046-021-02209-w. 34980213 PMC8722039

[91] Son B, Lee S, Kim H, Kang H, Jeon J, Jo S, Seong KM, Lee SJ, Youn H, Youn B. Decreased FBP1 expression rewires metabolic processes affecting aggressiveness of glioblastoma. Oncogene. 2020;39(1):36–49. doi: 10.1038/s41388-019-0974-4. 31444412

[92] Li H, Qi Z, Niu Y, Yang Y, Li M, Pang Y, Liu M, Cheng X, Xu M, Wang Z. FBP1 regulates proliferation, metastasis, and chemoresistance by participating in C-MYC/STAT3 signaling axis in ovarian cancer. Oncogene. 2021;40(40):5938–5949. doi: 10.1038/s41388-021-01957-5. 34363022 PMC8497274

[93] Li Y, Jiang B, Zeng L, Tang Y, Qi X, Wan Z, Feng W, Xie L, He R, Zhu H, et al. Adipocyte-derived exosomes promote the progression of triple-negative breast cancer through circCRIM1-dependent OGA activation. Environ Res. 2023;239(Pt 1):117266. doi: 10.1016/j.envres.2023.117266. 37775001

[94] García-Contreras M, Vera-Donoso CD, Hernández-Andreu JM, García-Verdugo JM, Oltra E, Ambrósio CE. Therapeutic potential of human adipose-derived stem cells (ADSCs) from cancer patients: a pilot study. PLOS ONE. 2014;9(11):e113288. doi: 10.1371/journal.pone.0113288. 25412325 PMC4239050

[95] Fang J, Rao X, Wang C, Wang Y, Wu C, Zhou R. Role of exosomes in modulating non-small cell lung cancer radiosensitivity. Front Pharmacol. 2024;15:1471476. doi: 10.3389/fphar.2024.1471476. 39737074 PMC11683128

[96] Sun Z, Shi K, Yang S, Liu J, Zhou Q, Wang G, Song J, Li Z, Zhang Z, Yuan W. Effect of exosomal miRNA on cancer biology and clinical applications. Mol Cancer. 2018;17(1):147. doi: 10.1186/s12943-018-0897-7. 30309355 PMC6182840

[97] Wang Z, Aguilar EG, Luna JI, Dunai C, Khuat LT, Le CT, Mirsoian A, Minnar CM, Stoffel KM, Sturgill IR, et al. Paradoxical effects of obesity on T cell function during tumor progression and PD-1 checkpoint blockade. Nat Med. 2019;25(1):141–151. doi: 10.1038/s41591-018-0221-5. 30420753 PMC6324991

[98] Hayes AJ, Larkin J. BMI and outcomes in melanoma: more evidence for the obesity paradox. The Lancet Oncol. 2018;19(3):269–270. doi: 10.1016/S1470-2045(18)30077-9. 29449191

[99] Batrakova EV, Kim MS. Using exosomes, naturally-equipped nanocarriers, for drug delivery. J Educ Chang Controlled Release: official Journal Of The Controlled Release Society. 2015;219:396–405. doi: 10.1016/j.jconrel.2015.07.030. PMC4656109 26241750

[100] Ailuno G, Baldassari S, Lai F, Florio T, Caviglioli G. Exosomes and extracellular vesicles as emerging theranostic platforms in cancer research. Cells. 2020;9(12):2569. doi: 10.3390/cells9122569. 33271820 PMC7761021

[101] Ebrahimian M, Hashemi M, Etemad L, Salmasi Z. Thymoquinone-loaded mesenchymal stem cell-derived exosome as an efficient nano-system against breast cancer cells. Iran J Basic Med Sci. 2022;25(6):723–731. doi: 10.22038/IJBMS.2022.64092.14116. 35949303 PMC9320205

[102] Shirzad M, Daraei A, Najafzadehvarzi H, Farnoush N, Parsian H. Co-culture system of breast cancer and normal cells to investigate inflammation: using doxorubicin encapsulated in adipose-derived exosomes. Med Oncol. 2024;42(1):21. doi: 10.1007/s12032-024-02568-2. 39630192

[103] Callegari E, D’Abundo L, Guerriero P, Simioni C, Elamin BK, Russo M, Cani A, Bassi C, Zagatti B, Giacomelli L, et al. miR-199a-3p modulates MTOR and PAK4 pathways and inhibits tumor growth in a hepatocellular carcinoma transgenic mouse model. Mol Ther Nucleic Acids. 2018;11:485–493. doi: 10.1016/j.omtn.2018.04.002. 29858083 PMC5992479

[104] Hou J, Lin L, Zhou W, Wang Z, Ding G, Dong Q, Qin L, Wu X, Zheng Y, Yang Y, et al. Identification of miRnomes in human liver and hepatocellular carcinoma reveals miR-199a/b-3p as therapeutic target for hepatocellular carcinoma. Cancer Cell. 2011;19(2):232–243. doi: 10.1016/j.ccr.2011.01.001. 21316602

[105] Sheykhhasan M, Kalhor N, Sheikholeslami A, Dolati M, Amini E, Fazaeli H. Exosomes of mesenchymal stem cells as a proper vehicle for transfecting miR-145 into the breast cancer cell line and its effect on metastasis. biomed Res Int. 2021;2021(1):5516078. doi: 10.1155/2021/5516078. 34307654 PMC8263260

[106] Zemanek T, Danisovic L, Nicodemou A. Exosomes and solid cancer therapy: where are we now? Med Oncol. 2025;42(3):77. doi: 10.1007/s12032-025-02626-3. 39961904 PMC11832697

[107] Yin H, Xie J, Xing S, Lu X, Yu Y, Ren Y, Tao J, He G, Zhang L, Yuan X, et al. Machine learning-based analysis identifies and validates serum exosomal proteomic signatures for the diagnosis of colorectal cancer. Cell Rep Med. 2024;5(8):101689. doi: 10.1016/j.xcrm.2024.101689. 39168094 PMC11384723

[108] Witwer KW. Minimal information for studies of extracellular vesicles 2023: relevance to cell and gene therapies. Cytotherapy. 2024;26(10):1119–1121. doi: 10.1016/j.jcyt.2024.05.018. 39046387

[109] Welsh JA, Goberdhan DCI, O’Driscoll L, Buzas EI, Blenkiron C, Bussolati B, Cai H, Di Vizio D, Driedonks TAP, Erdbrügger U, et al. Minimal information for studies of extracellular vesicles (MISEV2023): from basic to advanced approaches. J Extracell Vesicles. 2024;13(2):e12404. doi: 10.1002/jev2.12404. 38326288 PMC10850029

[110] Rhim WK, Kim JY, Lee SY, Cha SG, Park JM, Park HJ, Park CG, Han DK. Recent advances in extracellular vesicle engineering and its applications to regenerative medicine. Biomater Res. 2023;27(1):130. doi: 10.1186/s40824-023-00468-6. 38082304 PMC10712135

[111] Youssef E, Palmer D, Fletcher B, Vaughn R. Exosomes in Precision oncology and beyond: from bench to bedside in diagnostics and therapeutics. Cancers (basel). 2025;17(6):940. doi: 10.3390/cancers17060940. 40149276 PMC11940788

[112] Li J, Wang J, Chen Z. Emerging role of exosomes in cancer therapy: progress and challenges. Mol Cancer. 2025;24(1):13. doi: 10.1186/s12943-024-02215-4. 39806451 PMC11727182

[113] Erana-Perez Z, Igartua M, Santos-Vizcaino E, Hernandez RM. Genetically engineered loaded extracellular vesicles for drug delivery. Trends Pharmacol Sci. 2024;45(4):350–365. doi: 10.1016/j.tips.2024.02.006. 38508958

[114] Timofeeva AM, Paramonik AP, Sedykh SS, Nevinsky GA. Milk exosomes: next-generation agents for delivery of anticancer drugs and therapeutic nucleic acids. Int J Mol Sci. 2023;24(12):10194. doi: 10.3390/ijms241210194. 37373342 PMC10298983

[115] Huang X, Wu W, Jing D, Yang L, Guo H, Wang L, Zhang W, Pu F, Shao Z. Engineered exosome as targeted lncRNA MEG3 delivery vehicles for osteosarcoma therapy. J Control Release. 2022;343:107–117. doi: 10.1016/j.jconrel.2022.01.026. 35077741

[116] Lee KWA, Chan LKW, Hung LC, Phoebe LKW, Park Y, Yi KH. Clinical applications of exosomes: a critical review. Int J Mol Sci. 2024;25(14):7794. doi: 10.3390/ijms25147794. 39063033 PMC11277529

[117] Fujita M, Hatta T, Ikka T, Onishi T. The urgent need for clear and concise regulations on exosome-based interventions. STEM Cell Rep. 2024;19(11):1517–1519. doi: 10.1016/j.stemcr.2024.09.008. PMC11589178 39454583

[118] Lewis ND, Sia CL, Kirwin K, Haupt S, Mahimkar G, Zi T, Xu K, Dooley K, Jang SC, Choi B, et al. Exosome surface display of IL12 results in tumor-retained pharmacology with Superior potency and limited systemic exposure compared with recombinant IL12. Mol Cancer Ther. 2021;20(3):523–534. doi: 10.1158/1535-7163.MCT-20-0484. 33443094

[119] Chinnappan M, Srivastava A, Amreddy N, Razaq M, Pareek V, Ahmed R, Mehta M, Peterson JE, Munshi A, Ramesh R. Exosomes as drug delivery vehicle and contributor of resistance to anticancer drugs. Cancer Lett. 2020;486:18–28. doi: 10.1016/j.canlet.2020.05.004. 32439419 PMC7327711

